# Facile Galvanic Replacement Toward One-Dimensional Cu-Based Bimetallic Nanobelts

**DOI:** 10.3390/nano16010038

**Published:** 2025-12-26

**Authors:** Ying Xie, Qitong Sun, Yuanyuan Li, Wanwan Li, Zhiwei Hou, Lihui Wei, Sujun Guan

**Affiliations:** 1 School of Physics and Advanced Energy, Henan University of Technology, Zhengzhou 450001, China; 2 Guoneng Mengjin Thermal Power Co., Ltd., Luoyang 471112, China

**Keywords:** galvanic replacement, nanobelts, heterostructured nanomaterials, in situ growth

## Abstract

We report a galvanic replacement-driven strategy for the in situ growth of highly uniform one-dimensional (1D) Cu@CuO-X (X = Ag, Bi) nanobelts directly on aluminum foils. Unlike conventional multi-step coating or hard-template replication strategies, the formation of these heterostructured nanobelts is governed by a spontaneous interfacial galvanic replacement process between Cu and the introduced metal species, ensuring in situ growth and intimate interfacial integration. Comprehensive SEM, TEM, XRD, and XPS characterizations confirm the successful formation of Cu@CuO-Ag and Cu@CuO-Bi architectures, where Bi predominantly exists in the oxidized Bi^3+^ state, forming Bi_2_O_3_-like surface species. Benefiting from their 1D anisotropic framework and controllable heterointerfaces, this work underscores the distinctiveness and versatility of the self-templated galvanic replacement strategy for the design of multifunctional nanomaterials.

## 1. Introduction

Low-dimensional metal nanostructures, such as nanowires, nanorods, and nanotubes, have emerged as a distinct class of materials with unique geometric and physicochemical advantages [1,2,3]. Their large surface-to-volume ratios expose abundant active sites, thereby promoting interfacial reactions. In particular, the intrinsic anisotropy of one-dimensional (1D) nanostructures enables directional charge transport and enhanced light-matter coupling, giving rise to tunable electronic, plasmonic, and catalytic properties. Rational nanostructural design is therefore essential for effectively translating these intrinsic advantages into practical performance across a wide range of applications, including plasmon-enhanced sensing, energy conversion, optoelectronic devices, and catalysis [4,5,6,7,8,9,10,11,12,13,14,15].

Among various metal materials, copper (Cu)-based catalysts stand out owing to their abundance, low cost, excellent catalytic activity, and well-established morphology-regulation strategies [16,17,18,19,20,21,22,23]. In particular, the multiple valence states of Cu (Cu^0^, Cu^+^, and Cu^2+^) endow it with rich redox flexibility and diverse surface chemistry, enabling its participation in a wide range of chemical reactions, such as CO_2_ reduction [24,25,26,27,28,29,30,31,32]. However, despite the promising catalytic potential of Cu and Cu_x_O phases, their performance is limited by low intrinsic activity, poor stability, and competing hydrogen evolution reactions. To overcome these limitations, the incorporation of a secondary metal component has emerged as an effective strategy to modulate the local electronic structure, regulate atomic coordination, and enhance structural integrity [33,34,35,36]. Ag and Bi are especially attractive candidates. Ag preferentially facilitates CO formation, whereas Bi promotes formate generation, and their integration with Cu has been shown to improve product selectivity, reduce overpotentials, and enhance structural stability in bimetallic nanostructures [37,38].

Beyond composition, catalyst architecture plays a critical role in determining catalytic performance. One-dimensional Cu nanobelts provide continuous frameworks with large, exposed surface areas. To date, Cu nanobelts and related belt-like nanostructures have been synthesized via chemical reduction, surfactant-assisted growth and interfacial self-assembly approaches [39,40,41]. In contrast, galvanic replacement offers a distinct advantage for constructing nanostructures on self-supported substrates by enabling spontaneous redox-driven metal exchange accompanied by structural reconstruction under mild conditions [42]. Importantly, galvanic replacement allows the introduction of Ag or Bi while retaining the preformed nanobelt architecture, making it particularly suitable for constructing bimetallic Cu-based nanobelts with controlled composition and well-defined interfacial structures.

**Scheme 1:**
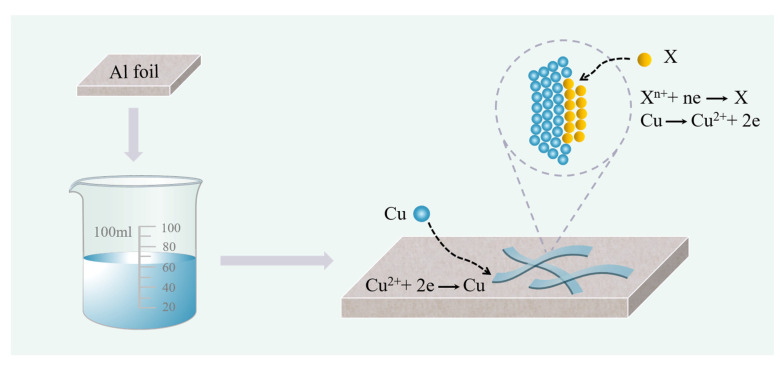
Schematic illustration of the synthesis process of Cu@CuO-X nanobelts (X = Ag, Bi) via a spontaneous galvanic-replacement strategy on an Al substrate.

Herein, we present a facile galvanic replacement strategy for the in situ fabrication of highly uniform 1D Cu@CuO-X (X = Ag, Bi) nanobelts on aluminum (Al) foil (Scheme 1). Comprehensive structural and compositional analyses confirm the successful formation of well-defined bimetallic heterostructures. The method establishes a general framework for directing self-regulated redox transformations toward structurally integrated heterostructures, offering distinct advantages in achieving controllable composition, coherent interfaces, and scalable synthesis with potential for future exploration in photocatalysis and related functional applications.

## 2. Materials and Methods

### 2.1. Chemicals and Materials

Copper (II) chloride dihydrate (CuCl_2_·2H_2_O, ≥99.0%), silver nitrate (AgNO_3_, ≥99.8%), bismuth (III) nitrate pentahydrate (Bi(NO_3_)_3_·5H_2_O, ≥98.0%), nitric acid (HNO_3_, ≥65%), phosphoric acid (H_3_PO_4_, ≥85%) were purchased from Sigma-Aldrich. (St. Louis, MO, USA). Hexadecyltrimethylammonium chloride (CTAC, 97%) was purchased from Aladdin Chemical Reagent Co., Ltd. (Shanghai, China). Al foil was purchased from Haochen Metal Materials Trading Company (Chaohu, China) with a thickness is 0.01 mm.

### 2.2. Preparation of Cu@CuO-X (Ag, Bi) Nanobelts

Cu@CuO-X (Ag, Bi) nanobelts were synthesized through a galvanic replacement process on aluminum foils.

For Cu@CuO-Ag nanobelts, CuCl_2_ (65 mg) was first dissolved in 50 mL of an aqueous CTAC solution (1.78 mM) containing HNO_3_ (12 μL) under stirring in a glass vial. Subsequently, AgNO_3_ (4.3 mg) was added, and the solution was stirred for 5 min to ensure homogeneous mixing. An Al foil (2.5 × 2.5 cm^2^) was pre-cleaned by immersion in 3 mL of aqueous H_3_PO_4_ for 2 min, rinsed thoroughly with deionized water, and immersed in the mixture at 5 °C without stirring. During this process, a spontaneous galvanic replacement reaction occurred between Cu^2+^ species and the Al substrate, leading to the formation of Cu-based nanobelts, followed by interfacial replacement between Cu and Ag^+^ ions. After 24 h, the Al foil was removed and rinsed with deionized water. For Cu@CuO-Bi nanobelts, the procedures were identical except that AgNO_3_ was replaced by Bi(NO_3_)_3_ solution (80 μL, 100 mM) as the precursor.

### 2.3. Sample Characterization

The morphology and structure of products were characterized by field-emission scanning electron microscopy (FESEM, Regulus 8100, Hitachi High-Tech, Tokyo, Japan, and Gemini SEM-300, ZEISS, Oberkochen, Germany) coupled with an energy dispersive spectrometer (EDS), as well as by transmission electron microscopy (TEM, JEM-2800, JEOL, Tokyo, Japan). X-ray diffraction (XRD) patterns were recorded using a Bruker D8 Advance diffractometer equipped with a Cu Kα X-ray source (Bruker, Karlsruhe, Germany). X-ray photoelectron spectroscopy (XPS) measurements were performed on an ESCALab 220XL spectrometer (VG Scientific, East Grinstead, UK) using 300 W Al Kα radiation.

## 3. Results and Discussion

**Figure 1:**
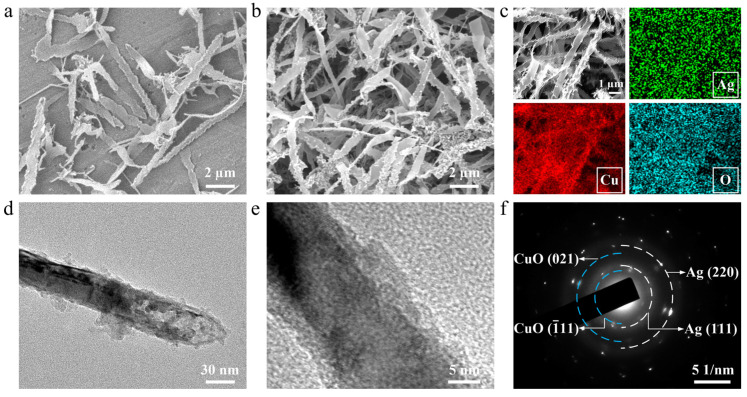
(**a**) SEM image of Cu nanobelts grown on Al foil. (**b**,**c**) SEM and EDS mapping images of Cu@CuO-Ag nanobelts. (**d**–**f**) TEM, HRTEM image and corresponding SAED pattern of Cu@CuO-Ag nanobelts.

Cu@CuO-Ag catalysts were synthesized via a galvanic replacement method, using CuCl_2_·2H_2_O and AgNO_3_ as precursors in an aqueous CTAC solution. Al foil was selected as the substrate due to its practical advantages and highly negative standard reduction potential, enabling spontaneous galvanic replacement reactions [43]. The morphology of the samples was characterized by scanning electron microscopy (SEM). As shown in Figure 1a, highly uniform 1D Cu nanobelts were distributed on the Al foil when CuCl_2_·2H_2_O was used as the precursor at 5 °C. In contrast, at room temperature, the relatively fast reaction kinetics favor rapid axial growth, resulting predominantly in the formation of Cu nanowires (Figure S1). Lowering the reaction temperature to 5 °C effectively slows the reaction kinetics and promotes lateral anisotropic growth, thereby leading to the formation of well-defined Cu nanobelts. When both CuCl_2_·2H_2_O and AgNO_3_ were employed, SEM images confirmed the preservation of the belt-like morphology (Figure 1b and Figure S2). The low-magnification SEM image (Figure S3) revealed uniform and continuous nanobelt coverage on the Al foil with only a small fraction of the substrate exposed, while the mass increase after synthesis (Δm ≈ 0.02 mg cm^−2^) indicated a high nanobelt loading. The nanobelts exhibited an average width of 577.1 nm, and lengths of up to 5 µm (Figure S4). EDS elemental mapping of Cu@CuO-Ag nanobelts revealed that Ag and O were uniformly distributed, whereas Cu was relatively dispersed (Figure 1c). In addition, Al signals were detected, which can be attributed to the Al foil substrate (Figure S5). TEM image revealed a belt-like morphology, consistent with SEM observations (Figure 1d). High-resolution TEM (HRTEM) image showed an approximately 1.0 nm-thick shell surrounding the nanobelts, which was attributed to surface oxidation of Cu upon exposure to dissolved oxygen (Figure 1e). Based on contrast features, the nanobelts were inferred to consist of a metallic Cu core encapsulated by a thin oxidized surface layer. To further investigate the phase composition and crystal structure of the outer layer, selected-area electron diffraction (SAED) pattern exhibited concentric, discontinuous rings rather than discrete single-crystal spots, indicating the presence of a polycrystalline shell (Figure 1f). Diffraction analysis revealed that the experimental patterns matched face-centered cubic Ag and monoclinic CuO. Simulated half-ring diffraction patterns were provided in Figure 1f for comparison. The measured d-spacings of 0.23 and 0.14 nm were assigned to Ag (111) and Ag (220), whereas 0.25 and 0.16 nm were indexed to CuO ($\bar{1}$11) and CuO (021). These results confirm the coexistence of metallic Ag and a thin CuO shell, with Ag embedded within the nanobelts.

**Figure 2:**
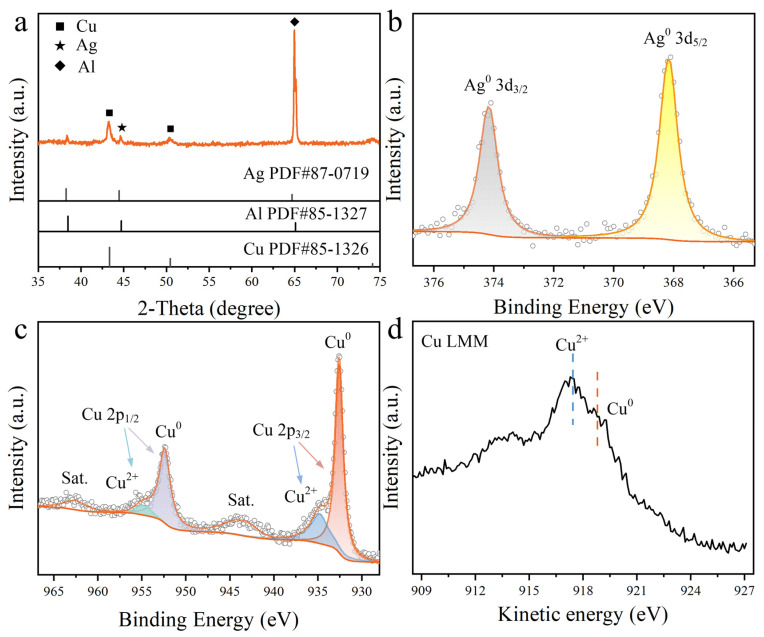
(**a**) XRD spectrum of Cu@CuO-Ag nanobelts. Standard XRD peaks for Cu, Ag, and Al are represented by the symbol “■”, “★”, and “◆”, respectively. (**b**–**d**) XPS spectra of Ag 3d (**b**), Cu 2p (**c**) and Cu LMM (**d**) for Cu@CuO-Ag nanobelts, respectively. Sat. refers to the satellite peaks characteristic of the Cu^2+^ oxidation state.

The XRD pattern showed the diffraction peaks at 43.3° and 50.4°, which were assigned to the (111) and (200) planes of Cu (PDF#85-1326), confirming the presence of the metallic Cu core (Figure 2a). An additional peak at 44.5° was indexed to the (200) plane of metallic Ag (PDF#87-0719). The diffraction peak at 65.1° was attributed to the (220) plane of the Al substrate (PDF#85-1327). No characteristic CuO diffraction peaks were observed, confirming the presence of an amorphous CuO surface layer [32]. XPS results further confirmed the presence of Ag, Cu, and O species. The Ag 3d XPS peaks at 368.2 eV and 374.2 eV were assigned to Ag^0^ species, consistent with the XRD results (Figure 2b). The Cu 2p spectrum was deconvoluted into six peaks, corresponding to the 2p_3/2_ and 2p_1/2_ states of Cu^0^, Cu^2+^, along with shake-up satellite peaks (Figure 2c). The Cu LMM Auger spectrum exhibited a dominant peak at 917.5 eV, characteristic of the oxidized Cu^2+^ state, and a weaker peak at 918.8 eV attributable to metallic Cu^0^ (Figure 2d). Considering that the Cu 2p signal contains contributions from both the outer surface and the near-surface bulk, whereas the Cu LMM transition is more surface-sensitive and primarily reflects the outermost atomic layers, these results indicate that the surface copper species are predominantly composed of CuO, while the underlying bulk of the nanobelts remains metallic Cu, consistent with the TEM observations.

**Figure 3:**
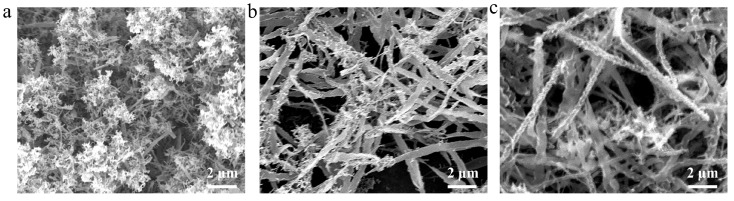
(**a**–**c**) SEM characterization of the nanostructures synthesized without HNO_3_ (**a**), with 12 µL HNO_3_ (**b**), and with 15 µL HNO_3_ (**c**).

The addition of HNO_3_ was found to be critical in regulating the galvanic displacement process. At low concentrations, HNO_3_ sustained acidic conditions and locally adjusted the acidity near the Cu surface, thereby promoting activation of the Cu surface. Under acidic conditions, HNO_3_ also suppressed Ag^+^ hydrolysis and Ag_2_O formation, thereby promoting selective Ag deposition on Cu and preserving the nanobelt-like morphology. The morphological evolution under varying HNO_3_ concentrations is shown in Figure 3. In the absence of HNO_3_, dendritic nanostructures were obtained. With increasing HNO_3_ concentration, well-defined nanobelts were obtained, highlighting the critical role of acidity in enabling controlled Ag deposition (Figure 3b,c).

**Figure 4:**
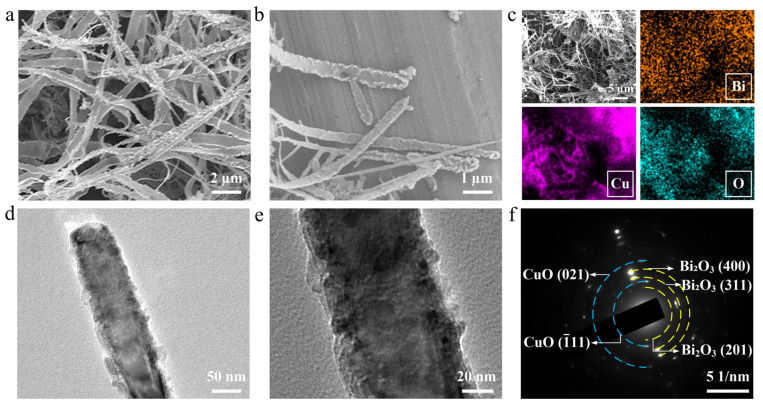
(**a**–**c**) SEM, enlarged SEM and EDS mapping images of Cu@CuO-Bi nanobelts. (**d**–**f**) TEM image, enlarged TEM image and corresponding SAED pattern of Cu@CuO-Bi nanobelts.

A similar synthetic strategy was employed to synthesize Cu@CuO-Bi belt-like nanostructures, with Bi(NO_3_)_3_ replacing AgNO_3_ as the precursor. SEM analysis revealed uniform 1D Cu@CuO-Bi nanobelts resembling those of Cu@CuO-Ag (Figure 4a,b and Figures S6 and S7). EDS elemental mapping showed the distribution of Bi, Cu, O and Al elements (Figure 4c and Figure S8). TEM and enlarged TEM images of individual Cu@CuO-Bi nanobelts revealed the presence of some nanoparticles decorating the surface (Figure 4d,e). The corresponding SAED pattern showed diffraction rings with d-spacings of 0.32 and 0.19 nm, which were assigned to the (201), (311), and (400) planes of Bi_2_O_3_. Additional rings at 0.25 and 0.16 nm were indexed to the ($\bar{1}$11) and (021) planes of CuO, confirming the coexistence of Bi_2_O_3_ and CuO (Figure 4f).

**Figure 5:**
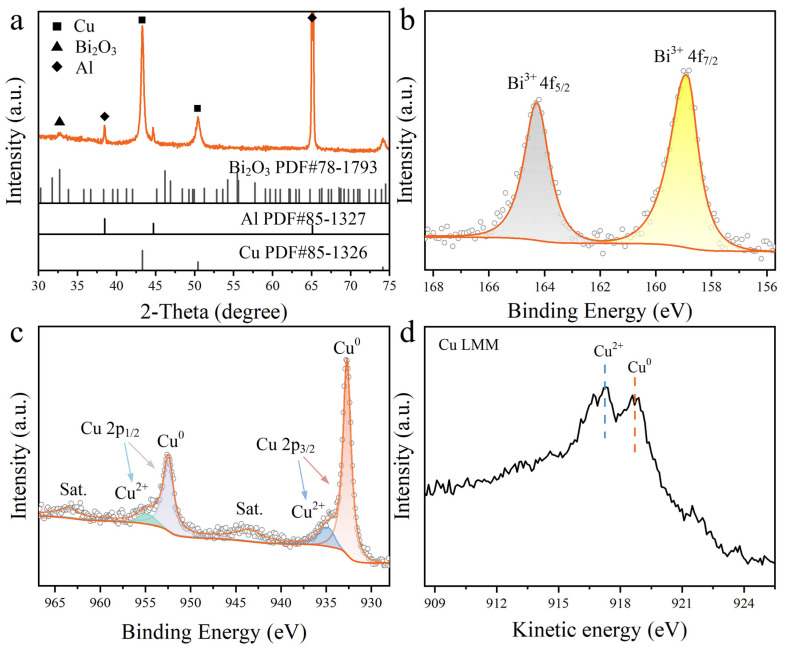
(**a**) XRD spectrum of Cu@CuO-Bi nanobelts. Standard XRD peaks for Cu, Bi_2_O_3_, and Al are represented by the symbol “■”, “▲”, and “◆”, respectively. (**b**–**d**) XPS spectra of Bi 4f (**b**), Cu 2p (**c**) and Cu LMM (**d**) for Cu@CuO-Bi nanobelts, respectively. Sat. refers to the satellite peaks characteristic of the Cu^2+^ oxidation state.

It is worth noting that in the XRD pattern of the Cu@CuO-Bi nanobelts (Figure 5a), a weak peak at 32.7°, indexed to the (220) planes of Bi_2_O_3_ (PDF#78-1793), was observed, consistent with the SAED results, which suggests that Bi_2_O_3_ likely forms at the interface. Similarly, the diffraction peaks from the Cu nanobelts and the Al substrate were also detected. The Bi 4f XPS peaks observed at 158.9 eV and 164.2 eV were also characteristic of Bi^3+^ species in the Cu@CuO-Bi nanobelts (Figure 5b). These results indicated that the displaced Bi was prone to oxidation into Bi_2_O_3_ and therefore predominantly existed in an oxidized state. Unlike Ag, which can remain in the metallic state and incorporate into the Cu lattice, Bi has a stronger thermodynamic tendency toward oxidation owing to the high stability of Bi–O bonds, forming surface oxide nanoparticles [33,34,35]. Meanwhile, Cu XPS for Cu@CuO-Bi was consistent with Cu@CuO-Ag, indicating comparable Cu oxidation states (Figure 5c,d). However, the Cu LMM Auger spectrum in Figure 5d exhibited a much more pronounced Cu^0^ feature, whereas that in Figure 2d was dominated by Cu^2+^, indicating that Cu@CuO-Bi nanobelts possess a thinner CuO layer and a more metallic Cu core than Cu@CuO-Ag nanobelts.

## 4. Conclusions

In conclusion, we have successfully developed a galvanic replacement-driven strategy for the in situ growth of highly uniform 1D Cu@CuO-X (X = Ag, Bi) nanobelts on aluminum foils. The formation of Cu@CuO-Ag and Cu@CuO-Bi heterostructures was confirmed through comprehensive characterization. This replacement reaction enables the direct substitution of various metals or materials, allowing the creation of diverse composite materials and heterostructures on the substrate, which, in turn, broadens the scope of potential applications. The resulting unique 1D anisotropic structure and tunable heterointerfaces further highlight the potential of this self-templated strategy for designing multifunctional nanomaterials, particularly for photocatalytic applications.

## Data Availability

The data presented in this study are available on request from the corresponding author after obtaining permission.

## Supplementary Materials

The following supporting information can be downloaded at https://www.mdpi.com/article/10.3390/nano16010038/s1.
