# Supplementary material for: Facile Galvanic Replacement Toward One-Dimensional Cu-Based Bimetallic Nanobelts
Source: Nanomaterials (Basel). 2025 Dec 26;16(1):38. doi: 10.3390/nano16010038 (PMC12787740; doi:10.3390/nano16010038)
Supplement: Supplementary file 1 [file nanomaterials-16-00038-s001.zip › nanomaterials-4030014-supplementary.pdf]

Supporting Information

Facile Galvanic Replacement toward One-Dimensional Cu-Based Bimetallic Nanobelts

Ying Xie[1]*, Qitong Sun[1], Yuanyuan Li[1], Wanwan Li[1], Zhiwei hou[1], Lihui Wei[2] and Sujun Guan[1]*

[1] School of Physics and Advanced Energy, Henan University of Technology, Zhengzhou 450001, China;

yxie_opt@haut.edu.cn

[2] Guoneng Mengjin Thermal Power Co., Ltd., Luoyang 471112, China;

[*] Correspondence: yxie_opt@haut.edu.cn (Y. Xie) tel: (+86)18623713850

guansujun@haut.edu.cn (S. J. Guan) tel: (+86)18623713382

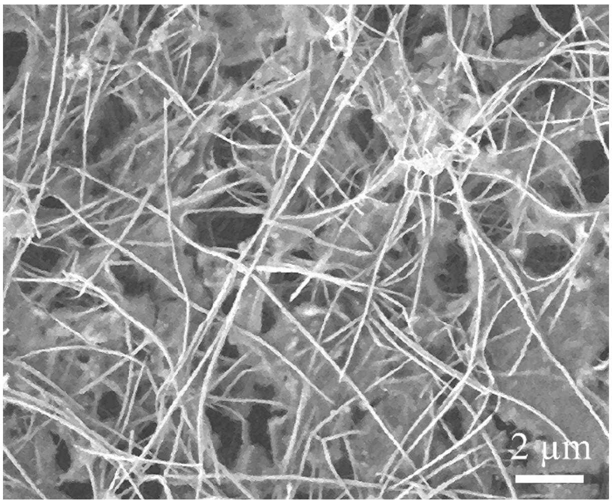

Figure S1. SEM image of Cu nanowires grown on Al foil at room temperature.

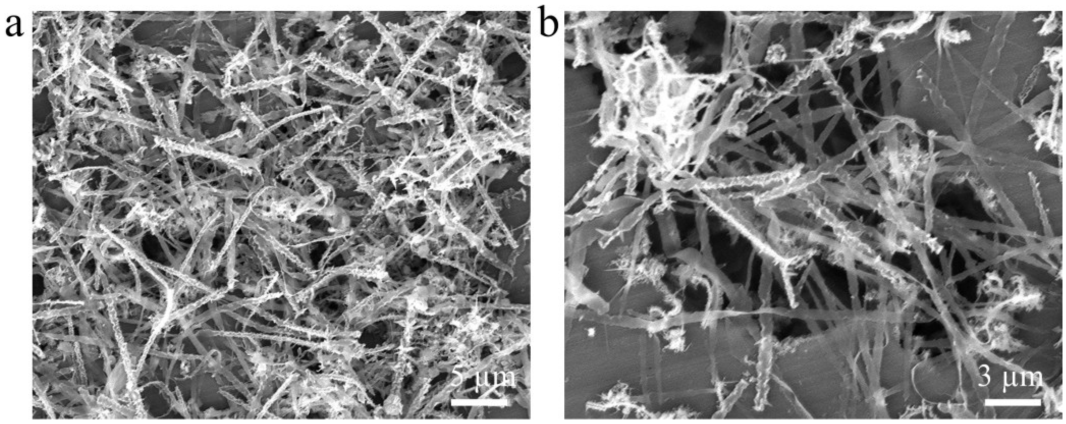

Figure S2. (a-b) Additional SEM images of Cu@CuO-Ag nanobelts grown on the Al foils.

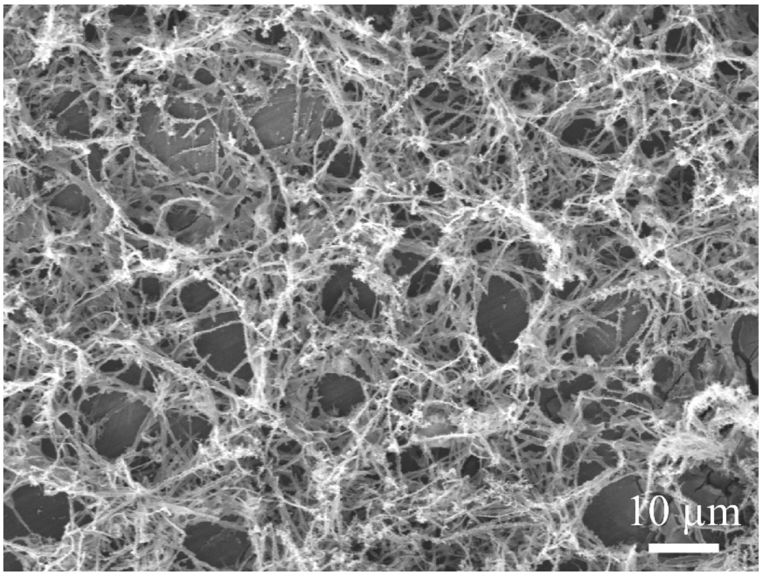

Figure S3. Low-magnification SEM image of Cu@CuO-Ag nanobelts grown on the Al foil.

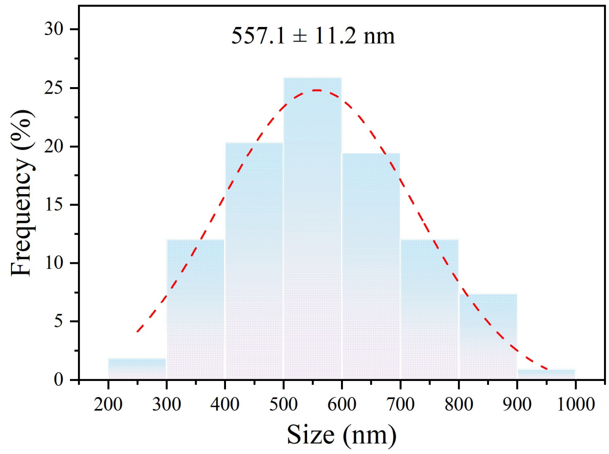

Figure S4. Size distribution histograms of Cu@CuO-Ag nanobelts.

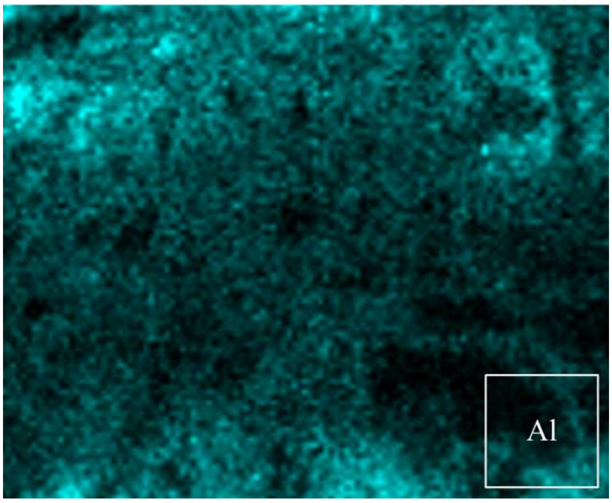

Figure S5. EDS mapping images of Al on Cu@CuO-Ag nanobelts.

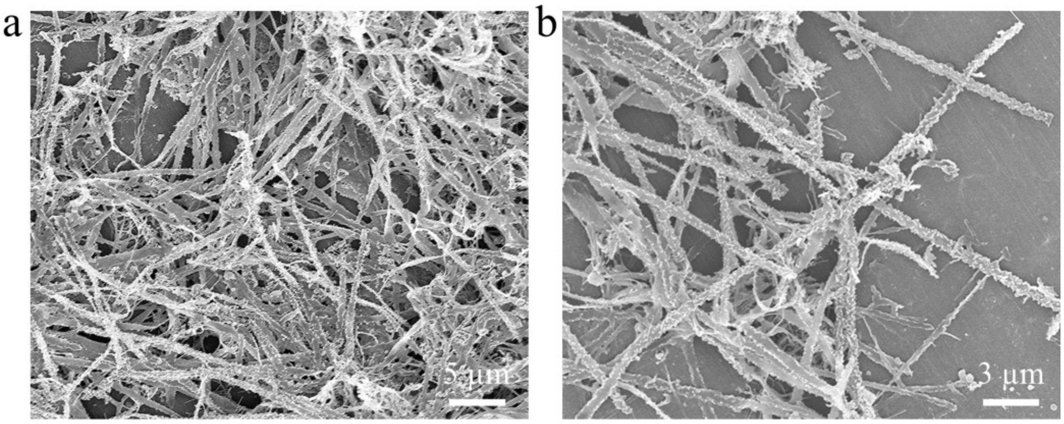

Figure S6. (a-b) Additional SEM images of Cu@CuO-Bi nanobelts grown on the Al foils.

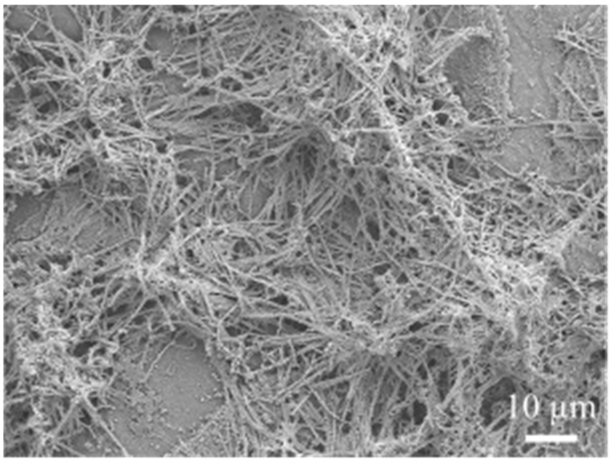

Figure S7. Low-magnification SEM image of Cu@CuO-Bi nanobelts grown on the Al foil.

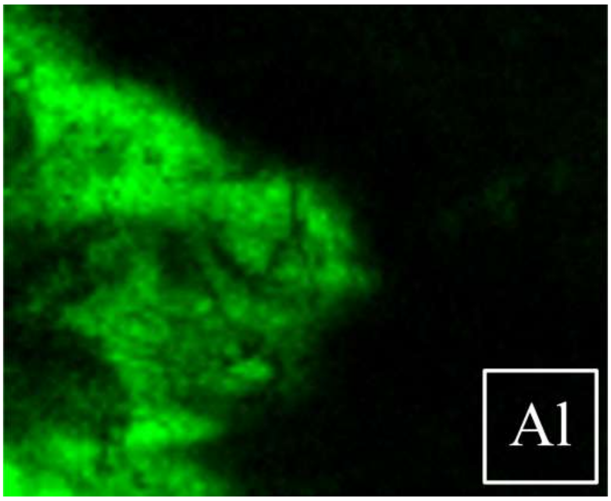

Figure S8. (a-b) EDS mapping images of Al on Cu@CuO-Bi nanobelts.
